# Automatic Path Tracking and Target Manipulation of a Magnetic Microrobot

**DOI:** 10.3390/mi7110212

**Published:** 2016-11-23

**Authors:** Jingyi Wang, Niandong Jiao, Steve Tung, Lianqing Liu

**Affiliations:** 1 State Key Laboratory of Robotics, Shenyang Institute of Automation, Chinese Academy of Sciences, Shenyang 110016, China; wangjingyi@sia.cn; 2 University of Chinese Academy of Sciences, Beijing 100049, China; 3 Department of Mechanical Engineering, University of Arkansas, Fayetteville, AR 72701, USA; chstung@uark.edu; 4 Beijing Advanced Innovation Center for Imaging Technology, Capital Normal University, Beijing 100037, China

**Keywords:** magnetic microrobot, automatic path tracking, expert control, magnetic microrobot, micromanipulation

## Abstract

Recently, wireless controlled microrobots have been studied because of their great development prospects in the biomedical field. Electromagnetic microrobots have the advantages of control agility and good precision, and thus, have received much attention. Most of the control methods for controlling a magnetic microrobot use manual operation. Compared to the manual method, the automatic method will increase the accuracy and stability of locomotion and manipulation of microrobots. In this paper, we propose an electromagnetic manipulation system for automatically controlling the locomotion and manipulation of microrobots. The microrobot can be automatically controlled to track various paths by using visual feedback with an expert control algorithm. A positioning accuracy test determined that the position error ranges from 92 to 293 μm, which is less than the body size (600 μm) of the microrobot. The velocity of the microrobot is nearly proportional to the applied current in the coils, and can reach 5 mm/s. As a micromanipulation tool, the microrobot is used to manipulate microspheres and microgears with the automatic control method. The results verify that the microrobot can drag, place, and drive the microstructures automatically with high precision. The microrobot is expected to be a delicate micromachine that could play its role in microfluidics and blood vessels, where conventional instruments are hard to reach.

## 1. Introduction

Because of their small size, high controllability, and mobility, wireless controlled microrobots have promising development prospects in the field of survey and manipulation for a microscale space such as microfluidic systems, blood vessels, and inner tissues [1]. To ensure that microrobots have a locomotion ability, various actuation methods have been developed such as electromagnetic [2,3,4,5,6,7], thermal [8], chemical bubble [9], swimming tail [10,11], bacterial [12,13], and hybrid [14]. Among these actuation methods, the electromagnetic actuation method has characteristic advantages. These include high controllability, high accuracy, and a large actuating force as a result of changing the currents in the electromagnetic coils. In addition, the low electromagnetic field intensity used to control the microrobots will not cause harm to, or have any negative side effects on, human beings.

However, the magnetic microrobots are mostly manually controlled through joystick or keyboard. The manual control method is an open-loop teleoperation in which the operator transmits motion information to the microrobots through an input device, without any feedback [15]. With this method, the microrobot can be controlled to move in a microfluidic chip [16], in 3D space [17], as well as in vivo [18]. However, manually controlled microrobots can only correct off-track motion if instructed by their operator, and the effectiveness of operation is highly dependent on the experience of the operator. Moreover, the automatic control method for the microrobot cannot completely replace the manual control method. We believe that the automatic and the manual method are two modes of operation and can be applied in different situations respectively. Additionally, the automatic and manual method can compensate for each other. For example, the manual control method is not appropriate for tasks that require high repetition with high precision [19,20,21].

To overcome the shortcomings of the manual control method for microrobots, automatic control is necessary and indispensable. The automatic method is a path-tracking close-loop control method with visual feedback to transmit the positional information to the close-loop controller; by regulating the electromagnetic force in real time, the error between the microrobot and its target position can be eliminated. In the case of manipulating microrobots in vivo, the microrobots will have difficulties with the presence of perturbations, boundary effects, and so on. For example, in the condition of existing external disturbance such as uneven liquid surface or flowing liquid, the automatic control method can operate the microrobot with greater accuracy. Meanwhile, it is very difficult to operate the microrobot to do the repetitive motion with the manual method. Hence, with the automatic control method, the motion of the microrobot will be more robust with high repetition and high precision.

In this paper, we propose the expert control algorithm for microrobot control with a novel electromagnetic manipulation system. Compared with proportional–integral–derivative (PID) controller [22,23], the expert controller has an advantage of self-adaption. Without testing the parameters of *P*, *I* and *D*, the expert controller can control microrobots with different sizes or magnetization. Also, with the expert control method, the driving force is reducing when the microrobot is nearing the target point. The tracking path is separated to several segments with target points. The moving speed is controlled to reduce to zero when the microrobot arrives at every target point. With the automatic control method using an expert control algorithm, the microrobot performs a point-by-point path-tracking motion. The predefined trajectory is divided into a series of adjacent points, then the microrobot tracks the points one by one to complete the path tracking. The microrobot can be controlled automatically to propel in the desired direction or track complex paths through image processing and a visual feedback control system. Furthermore, as a micromanipulation tool, the microrobot is used to drag, place, and rotate the micromachines automatically with high precision.

## 2. Methods

### 2.1. Theoretical Background of Magnetic Microrobot

For the alignment of the microrobot on the *xy* plane, the generated magnetic field (B) and the following torque (T) is generated as:

(1) $$T=VM\times B=V\left[\begin{array}{c} M_{x}\\ M_{y} \end{array}\right]\times \left[\begin{array}{c} B_{x}\\ B_{y} \end{array}\right]=V\left[\begin{array}{c} M_{x}\\ M_{y} \end{array}\right]\times \left[\begin{array}{c} B\cos\theta \\ B\sin\theta \end{array}\right]$$

where V, M, and B denote the volume and magnetization vector of the microrobot and the magnetic flux vector of the external magnetic field on the *xy* plane, respectively. The alignment direction (θ) of the microrobot is determined as tan $\theta =\tan\frac{B_{y}}{B_{x}}$.

When manipulating the microrobot in fluidic environment, the viscous drag force is the major resistance force for transport. In order to manipulate the microrobot, the input magnetic force must overcome the resistance:

(2) $$F\geq F_{d}$$

where F is the magnetic force exerted on the microrobot, and *F_d_* is the resistance of the fluid. The magnetic force is given by [24]:

(3) F=V(M⋅∇)B

where V is the volume of the microrobot, M is the magnetization vector of the microrobot, and B is the magnetic flux vector of the external magnetic field.

When the microrobot is actuated on the *xy* plane, ∇ is the gradient operator:

(4) $$\nabla =\left[\begin{array}{c} \frac{\partial }{\partial x}\\ \frac{\partial }{\partial y} \end{array}\right]$$

and the uniform magnetization of the microrobot and the magnetic flux are formed:

(5) $$M=\left[\begin{array}{c} M_{x}\\ M_{y} \end{array}\right],B=\left[\begin{array}{c} B_{x}\\ B_{y} \end{array}\right]$$

The magnetic force can be expressed in the following vector form:

(6) $$F=\left[\begin{array}{c} F_{x}\\ F_{y} \end{array}\right]=V\left[\begin{array}{cc} \frac{\partial B_{x}}{\partial x} & \frac{\partial B_{y}}{\partial x}\\ \frac{\partial B_{x}}{\partial y} & \frac{\partial B_{y}}{\partial y} \end{array}\right]\left[\begin{array}{c} M_{x}\\ M_{y} \end{array}\right]\approx V\left[\begin{array}{cc} \frac{\partial B_{x}}{\partial x} & 0\\ 0 & \frac{\partial B_{y}}{\partial y} \end{array}\right]\left[\begin{array}{c} M_{x}\\ M_{y} \end{array}\right]=V\left[\begin{array}{c} M_{x}\frac{\partial B_{x}}{\partial x}\\ M_{y}\frac{\partial B_{y}}{\partial y} \end{array}\right]$$

Compared with $\frac{\partial B_{x}}{\partial x}$ and $\frac{\partial B_{y}}{\partial y}$, $\frac{\partial B_{y}}{\partial x}$ and $\frac{\partial B_{x}}{\partial y}$ have very small values: ~3% of $\frac{\partial B_{x}}{\partial x}$ and $\frac{\partial B_{y}}{\partial y}$ [25]. $M_{x}$ and $M_{y}$ are in same order of magnitude, hence the cross-term of the force matrix may be ignored and are set to zero.

The magnetic field generated by the current flows through a spiral electromagnetic coil, and can be calculated by the Biot–Savart law as follows [26]:

(7) $$B=\frac{\mu_{0}I}{4\pi }\int_{L}\frac{dl\times h}{{\left|h\right|}^{3}}$$

where *I* is the current through the coil, *L* is the integral path, h is the full displacement vector from the wire element to the point where the field needs to be calculated, dl is a vector of the differential element of the current through the wire, and μ_0_ is the magnetic permeability (μ_0_ = 4π × 10^−7^ H/m).

The magnetic fields generated by the coils at the arbitrary position (*x*, *y*) can be described as the multiplication of the electromagnetic field value per unit current $\hat{B}(x,y)$ and the current (I) applied through each coil:

(8) $$B(x,y)=\hat{B}(x,y)I$$

The magnetic flux can be expressed as the function of the current I:

(9) $$\left[\begin{array}{c} B_{x}\\ B_{y} \end{array}\right]=\left[\begin{array}{c} \begin{array}{cccc} {\hat{B}}_{x,1} & {\hat{B}}_{x,2} & {\hat{B}}_{x,3} & {\hat{B}}_{x,4} \end{array}\\ \begin{array}{cccc} {\hat{B}}_{y,1} & {\hat{B}}_{y,2} & {\hat{B}}_{y,3} & {\hat{B}}_{y,4} \end{array} \end{array}\right]\left[\begin{array}{c} I_{1}\\ I_{2}\\ I_{3}\\ I_{4} \end{array}\right]$$

where $B_{x}(I_{n})$ and $B_{y}(I_{n})$ (*n* = 1, 2, 3, 4) denote the *x*-axis and *y*-axis directional magnetic fields generated by the n-th coils. The magnetic force can be expressed as:

(10) $$\begin{array}{l} F=\left[\begin{array}{c} F_{x}\\ F_{y} \end{array}\right]=V\left[\begin{array}{cc} \frac{\partial B_{x}}{\partial x} & \frac{\partial B_{y}}{\partial x}\\ \frac{\partial B_{x}}{\partial y} & \frac{\partial B_{y}}{\partial y} \end{array}\right]\left[\begin{array}{c} M_{x}\\ M_{y} \end{array}\right]=V\left[\begin{array}{c} M_{x}\frac{\partial B_{x}}{\partial x}\\ M_{y}\frac{\partial B_{y}}{\partial y} \end{array}\right]\\ =V\left[\begin{array}{c} \begin{array}{cccc} M_{x}\frac{\partial {\hat{B}}_{x,1}}{\partial x} & M_{x}\frac{\partial {\hat{B}}_{x,2}}{\partial x} & M_{x}\frac{\partial {\hat{B}}_{x,3}}{\partial x} & M_{x}\frac{\partial {\hat{B}}_{x,4}}{\partial x} \end{array}\\ \begin{array}{cccc} M_{y}\frac{\partial {\hat{B}}_{y,1}}{\partial y} & M_{y}\frac{\partial {\hat{B}}_{y,2}}{\partial y} & M_{y}\frac{\partial {\hat{B}}_{y,3}}{\partial y} & M_{y}\frac{\partial {\hat{B}}_{y,4}}{\partial y} \end{array} \end{array}\right]\left[\begin{array}{c} I_{1}\\ I_{2}\\ I_{3}\\ I_{4} \end{array}\right] \end{array}$$

From Equation (10), the magnetic force, the magnetic flux, and the gradient magnetic fields used to propel the microrobot in any position, can be calculated using the current in every coil. Therefore, the microrobot can be controlled to move to the desired position by changing the current in the electromagnetic coils.

### 2.2. Close-Loop Control of the Microrobot

**Figure 1:**
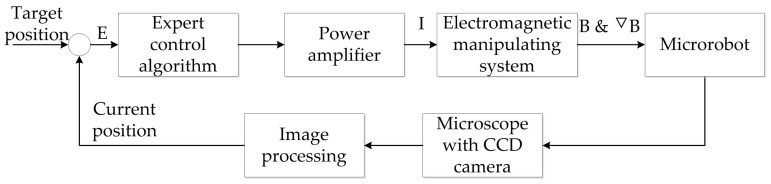
Structure diagram of the visual feedback control of the microrobot. The error between the real-time and target positions of the microrobot is set to be the input variable. *B* and ∇*B* are the generated electromagnetic field, and the gradient field to control the microrobot, respectively.

The microrobot can be actuated automatically by using visual feedback control. In order to actuate the microrobot to propel along a desired path accurately, a close-loop system using image processing and the expert control algorithm is set up. The structure diagram of the visual feedback expert control is shown in Figure 1.

First, the location information of the microrobot is identified by image recognition.

Second, the target position is determined. Then, the distance and desired alignment angle between the microrobot and the target position are calculated, where these are set to be the input variables. The expert controller can be expressed as

*U* = *f*(*E*, *I*, *K*)

where *U* is the output variable, *E* is the input set (distance and alignment angle between the microrobot and the target position), *I* is the output of the inference, *K* the knowledge item, and *f* is the intelligent operator of Expert Control. The principle of *f* is

If *E* and *K* then <If *I* then *U*>

where *I* is the result of the inference of input *E* and knowledge *K*. Then, the required control behavior is exported according to *I*. The method of knowledge representation is genetic rule. The output of the inference (*I*) is generated through the input (*E*) and knowledge and experience (*K*). Then, the output (*U*) is generated according to the output of the inference.

Third, the intelligent operator *f* is the relational operator between the input variables and the gradient magnetic fields generated by the unit current of each coil. The magnetic fields can be calculated from Equation (9).

The method of knowledge representation is genetic rule.

The output of the inference (*I*) is generated through the input (*E*) and knowledge and experience (*K*). Then, the output (*U*) is generated according to the output of the inference.

For example, the distance between microrobot and the target point is calculated as input (*E*). The output (*U*) is the current for the electromagnetic coils. The inference (*I*) is set as:

If the distance (*E*) is zero, then set the output as zero.

If the distance (*E*) is small (but not 0), then set the output as small.

If the distance (*E*) is large, then set the output as large.

The expression of zero, small, and large of distance can be expressed as EZ, ES, and EL.

Based on the knowledge and experience (*K*), EZ, ES, and EL can be expressed as:

EZ: *E* < 5 pixels

ES: 5 pixels ≤ *E* ≤ 500 pixels

EL: *E* > 500 pixels

Then, inference can be expressed as:

If EZ, then *U* = 0;

If ES, then *U* = *f*(*E*);

If EL, then *U* = MAX;

*f*(*E*) can be set as a liner function of *E*. Or *f*(*E*) can be set as a piecewise function. It can be adjusted according to the control precision. MAX can be set as a constant to control the microrobot at express speed.

Using the expert control algorithm, microrobot is a point-by-point path-tracking motion. With the expert control method, the driving force is reducing when the microrobot is nearing the target point. The tracking path is separated to several segments with target points. The moving speed is controlled to reduce to zero when the microrobot arrives at every target point. This control method can meet the moderate requirements of the control process for the quickness, stability, and non-overshoot.

**Figure 2:**
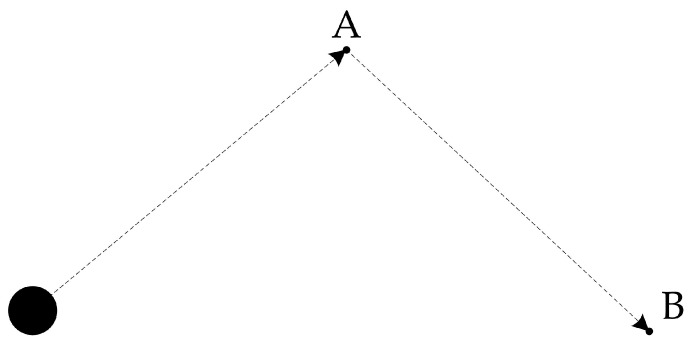
Schematic of expert control algorithm used in microrobot control.

The function of knowledge, *K*, used in our algorithm, is to change the magnitude and direction of the electromagnetic force, according to the distance between the real-time and the target positions of the microrobot. As shown in Figure 2, when the target points are set as point A and point B, the distance between the microrobot and point A is calculated first. Then, an electromagnetic force is generated to drive the microrobot to move to point A. This force decreases as the distance reduces, and the driving force falls to zero when the microrobot arrives at point A. Then, the distance between point A and point B is recalculated and the electromagnetic force is regenerated. This algorithm makes the microrobot move accurately with higher precision, smaller overshoot, and more robustness.

## 3. Experiments

### 3.1. Theoretical Background of Magnetic Microrobot

The electromagnetic manipulation microrobot was fabricated with NdFeB magnetic powder and polydimethylsiloxane (PDMS). The NdFeB powder has a high magnetization value, which can increase the propulsion force of the microrobot.

**Figure 3:**
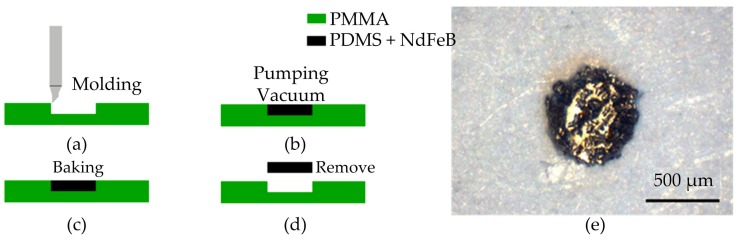
Process of microrobot fabrication. (**a**) Curving the microrobot mold on an acrylic plate; (**b**) putting the microrobot into the vacuum chamber to remove bubbles; (**c**) baking the microrobot for 4 h at 60 °C; (**d**) removing the microrobot from the mold; (**e**) top view of the microrobot.

The brief fabrication procedure and the microrobot are shown in Figure 3. First, the mold for the microrobot was designed as a thin disk. The diameter and the thickness of the microrobot are 600 and 200 μm, respectively. Second, the mold was fabricated using an engraving machine (Benchtop Engravers EGX-600/400, Roland DGA Corporation, Irvine, CA, USA) equipped with a milling cutter with a diameter of 200 μm. The mold was carved on an acrylic plate. Third, the PDMS and NdFeB magnetic powder were mixed at a weight ratio of 1:1. In order to make the PDMS and curing agent sufficient and even-mixing, the PDMS and curing agent were mixed first for about 5 min, and then the magnetic powder was added. Finally, the mixture was stirred for 30 min. Fourth, the mixture was put into the mold and the redundant mixture was removed. The microrobot was degassed in a vacuum box to remove bubbles, and then placed in the oven to bake at 60 °C for 4 h. Finally, the microrobot was removed from the mold with tweezers after it was cured.

### 3.2. Design of the Electromagnetic Manipulation System

**Figure 4:**
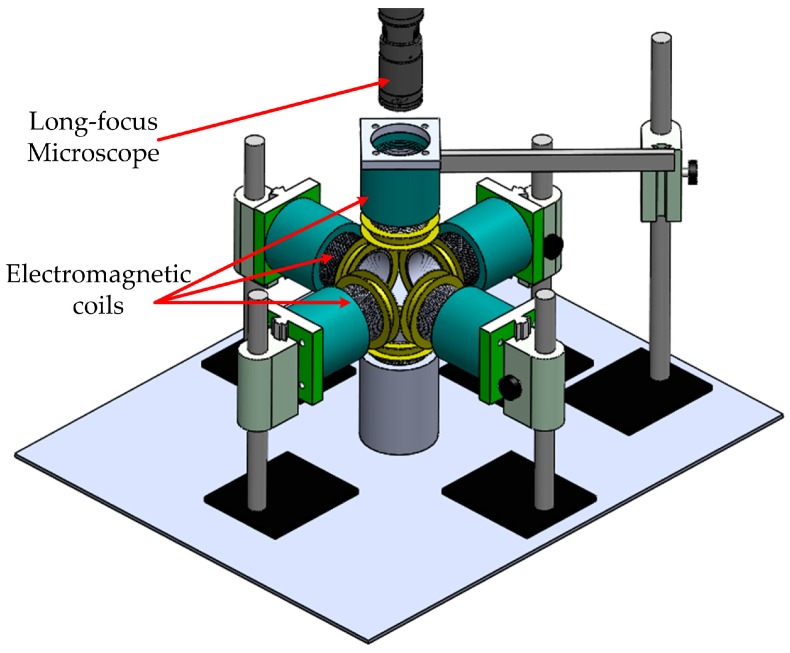
Schematic diagram of the electromagnetic manipulation system. There are six position-adjustable electromagnetic coils to adjust the manipulation region. Each electromagnetic coil is supported by the nylon plastic frames, which were designed as forward and backward precession. The height-adjustable pillar and the nylon plastic frames are convenient to adjust the vertical and horizontal position of the electromagnetic coils, respectively.

As shown in Figure 4, there are six electromagnetic coils in the electromagnetic manipulation system to manipulate the microrobot in three dimensions. Two of the pairs of the electromagnetic coils were set to be orthogonal in the horizontal plane, whereas the other pair was set in the vertical plane. The horizontal electromagnetic coils were installed on the height-adjustable pillar. Every electromagnetic coil is supported by the nylon plastic frames, which were designed for forward and backward precession. The height-adjustable pillar and the nylon plastic frames are convenient to adjust the vertical and horizontal position of the electromagnetic coils, respectively. The manipulation region of the system is limited as a result of the constant structure. To overcome this disadvantage, we propose a novel electromagnetic manipulation system that has position-adaptable electromagnetic coils. By regulating the six-position adaptable electromagnetic coils, the manipulation area can be adjusted to expand or shrink. A long-focus microscope with a charge-coupled device (CCD) was used to observe and provide visual feedback through the upper electromagnetic coil in the *z* direction.

**Figure 5:**
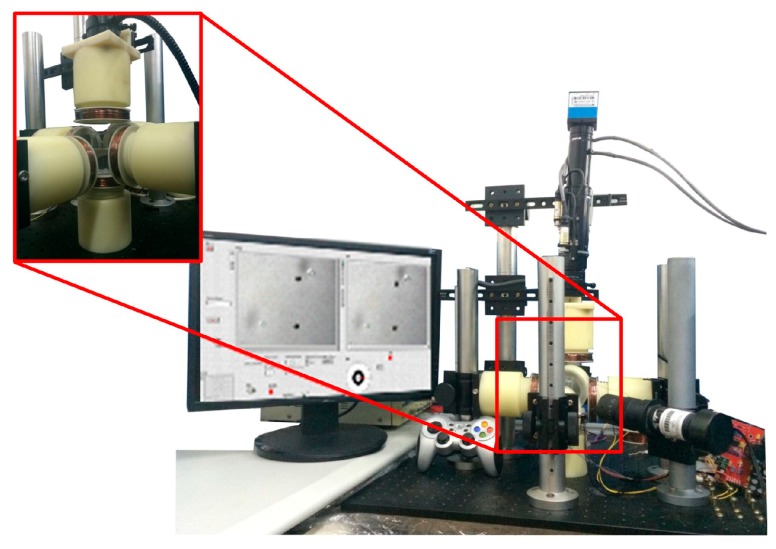
The whole electromagnetic manipulation system.

The actual electromagnetic manipulation system used is shown in Figure 5. It consists of an array of iron-core electromagnetic coils, a motorized long-focus microscope (Navitar 1-62317, Navitar Inc., New York, NY, USA), two data acquisition cards (NI PCI-6229 DAQ Card, National Instruments Corporation, Austin, TX, USA), a direct current (DC) power supply, a micron 3D positioning stage, and a power amplifier. The 3D positioning stage is used to adjust the position of the microrobot in the container.

The electromagnetic coils are supported by nylon plastic frames, which will not affect the magnetic field as a result of their nonmagnetic conductive property. The magnetic field for unit current of the electromagnetic coils is 2871 A/m, and the magnetic gradient field for unit current is 95726 A/m^2^. The detailed specification of the electromagnetic coils is described in Table 1.

In order to manipulate the microrobot, the location information is acquired by the electrical forcing microscope. The control algorithm, programmed by LabVIEW (Labview 2012, National Instruments Corporation), processes this information, generates the required output signal, and then transmits it to the data acquisition card. The required output current values, amplified by the power amplifier, are applied to the electromagnetic manipulating system to generate the required magnetic field. The microrobot is placed in a cubic container with deionized (DI) water on the micron 3D positioning stage in the center of the electromagnetic coils. The electromagnetic manipulation system can be used to observe and accurately manipulate the microrobot manually, as our previous work [16], or automatically.

## 4. Results

### 4.1. Velocity Measurement

In order to measure the velocity of the microrobot, various magnetic gradient fields were applied. These fields can be changed by the applied current through the coils.

**Figure 6:**
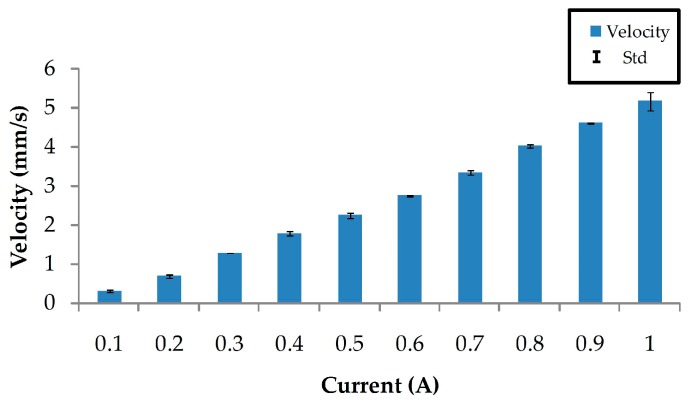
Velocity measurement of the microrobot according to the current through the electromagnetic coils.

The microrobot was placed on the surface of DI water. Initially, it started to accelerate from its stationary state due to the magnetic force. With the rapid increase in microrobot speed, the fluid viscous force was instantaneously increased. The microrobot can instantaneously reach a uniform velocity in a fluidic environment [27]. Five experiments were conducted at each applied current, and the average velocities were calculated. As shown in Figure 6, the velocity of the microrobot increased with an increase in applied current in the coils.

From the experimental results, we find that the velocity of the microrobot is approximately proportional to the applied current in the coils. When the applied current is 1 A, the velocity of the microrobot can be greater than 5 mm/s.

The velocity in the tracking process is not permanent. The microrobot is first accelerated and then moves with a constant velocity. Finally, it decelerates gradually. In order to track the path accurately, we set this initial driving current at 0.5 A (this means the initial velocity is set at about 2 mm/s).

### 4.2. Performance of the Close-Loop Control

**Figure 7:**
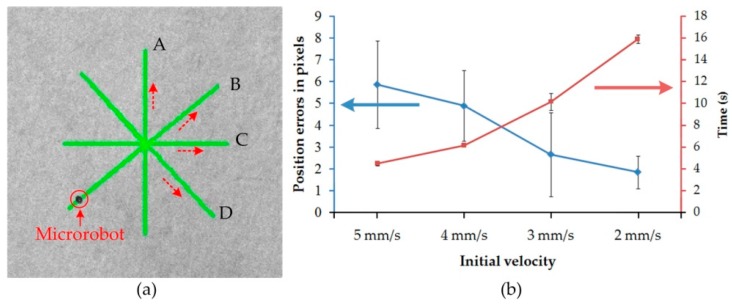
Position error measurement of microrobot. (**a**) Tracking lines (14 mm) for position error measurement; (**b**) position error measurement in pixels and average time versus different initial velocities.

The position accuracy of the microrobot is an important characteristic for close-loop control. The microrobot is controlled to track four paths (A, B, C, D) to evaluate the control accuracy, as shown in Figure 7. The permissible error affects the performance of the control. Based on a number of previous experiments, close-loop control of the microrobot is characterized by high accuracy, good stability, and rapid response when the permissible error range is set at 10 pixels. Therefore, the permissible error range is set at 10 pixels, which corresponds to 500 μm in length. This is less than the diameter of the microrobot.

As shown in Figure 7a, four equilong straight paths (A, B, C, D, 14 mm) were established for the microrobot to track. The microrobot was propelled along these paths, back and forth, with an automatic control method. Then, the position errors were calculated to measure the control performance. With different initial velocities (5 mm/s, 4 mm/s, 3 mm/s, 2 mm/s), each path is tracked three times, and the errors and average time are shown in Figure 7b. The position errors of the tracking path range from 92 to 293 μm. Consequently, the errors are all within the preset threshold value, and smaller than the half-size of the microrobot. As shown in Figure 7b, the position errors become bigger as the initial velocity increases. The time that the microrobot completes the straight line increases when the initial velocity increases. Hence, the microrobot has a good position accuracy when the initial velocity is set at a small value. In order to perform great accuracy, the initial velocity is set at small initial velocity when the microrobot performs close-loop tracking.

### 4.3. Close-Loop Tracking Performance of Microrobot

**Figure 8:**
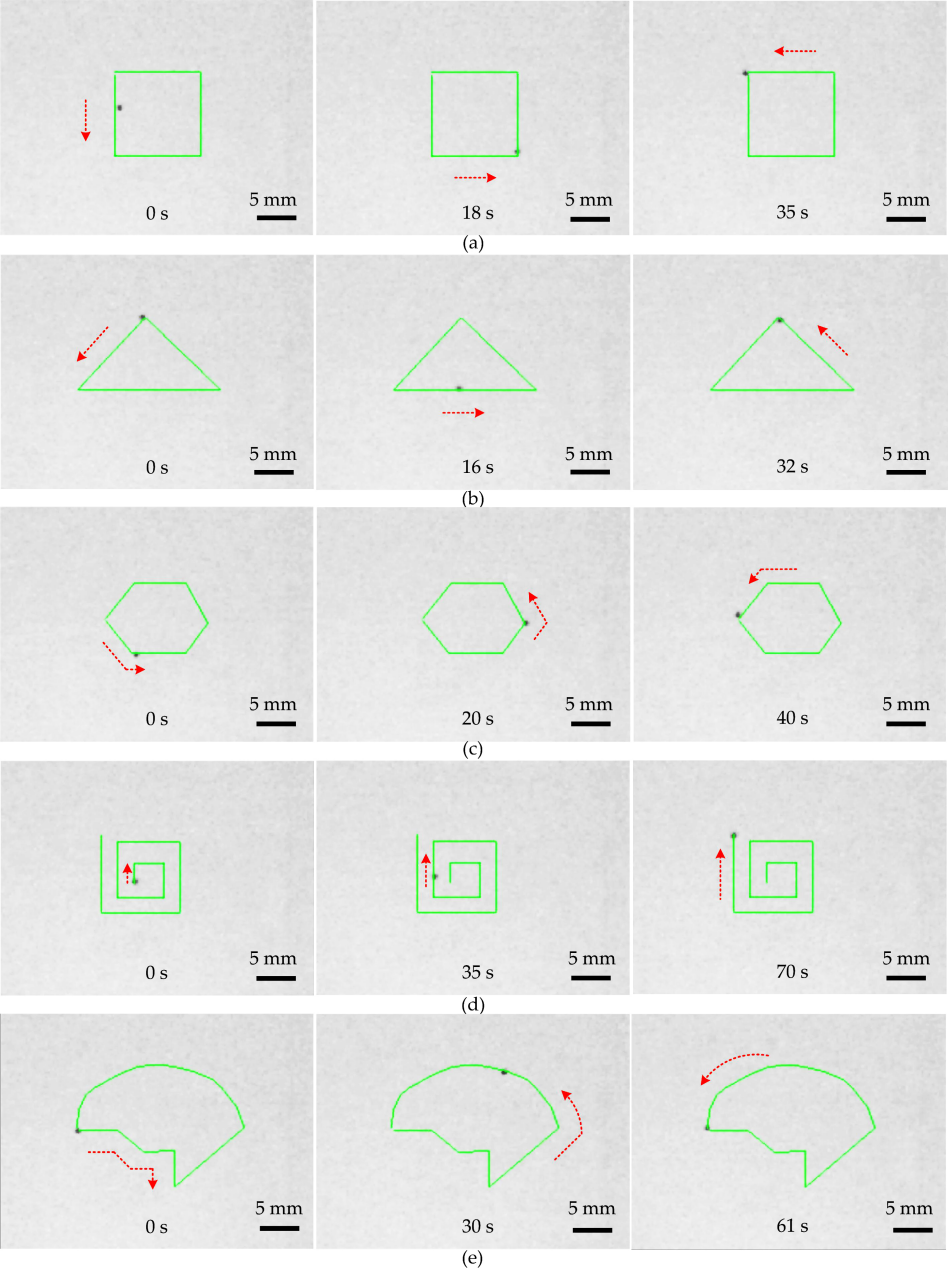
The expert control tracking performance of the microrobot. The green paths are set for the microrobot to track. The movement directions of the microrobot are marked in red dotted lines. The microrobot tracks the rectangular path (**a**); triangular path (**b**); hexagonal path (**c**); square spiral path (**d**); and complicated customized path (**e**). The scale bar is 5 mm.

Based on the expert control algorithm using visual feedback, a number of experiments regarding tracking on different paths were executed. First, the microrobot was recognized and the position information was acquired. Then, different paths were drawn for the microrobot tracking: rectangular, triangular, hexagonal, square spiral, and customized paths. When these are determined, the microrobot can accurately propel along the desired paths automatically. As shown in Figure 8, the green paths were designed as the desired paths for the microrobot. The red arrow indicates the direction of movement. The movements of the microrobot along the rectangular, triangular, hexagonal, square spiral, and customized paths takes approximately 35, 32, 40, 70 and 61 s, respectively.

### 4.4. Manipulating Microspheres

**Figure 9:**
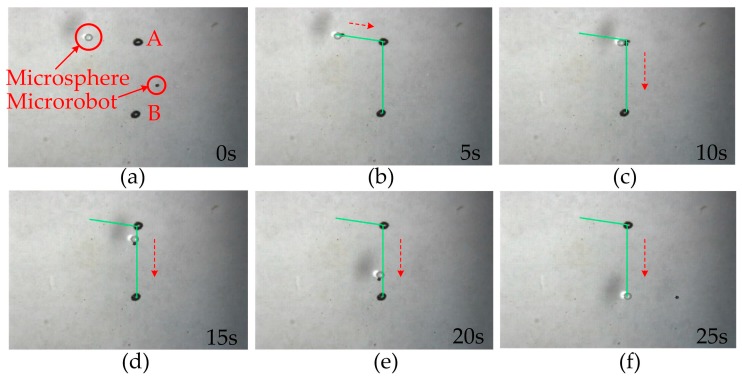
Manipulation of the microrobot with one microsphere. The microrobot and zirconia microsphere are marked in red circles. The green paths are set for the microrobot to track. The movement directions of the microrobot are marked by red dotted arrow lines. (**a**) The zirconia microspheres are placed on the liquid surface at random. (**b**) The microrobot drags the microsphere and moves to A point automatically. On arrival at A point (**c**), it drags the microsphere (**d**) and moves to B point automatically (**e**). Then, it is controlled to accelerate and separate from the microsphere (**f**). Then, it leaves the zirconia microsphere at point B. The manipulation procedure takes approximately 25 s.

The microrobot can be controlled to manipulate zirconia microspheres on the liquid surface. The diameter of the microsphere is approximately 1 mm, and it is placed on the liquid surface at random. The microrobot is controlled to propel towards the microsphere, and when it makes contact, it attaches to the microsphere as a result of the tensile force. As shown in Figure 9, for easily observation two points (A and B) are marked on the substrate, and the distance between them is 10 mm. In Figure 9, the microrobot is controlled to move towards the microsphere (a). The microrobot drags the microsphere and moves to A point automatically (b). On arrival at A point (c), it drags the microsphere (d) and moves to B point automatically (e). Then, it is controlled to accelerate and separate from the microsphere (f). The green paths are set for the microrobot to track. The movement directions are marked by red dotted arrow lines. The procedure of manipulation with one microsphere takes approximately 25 s.

**Figure 10:**
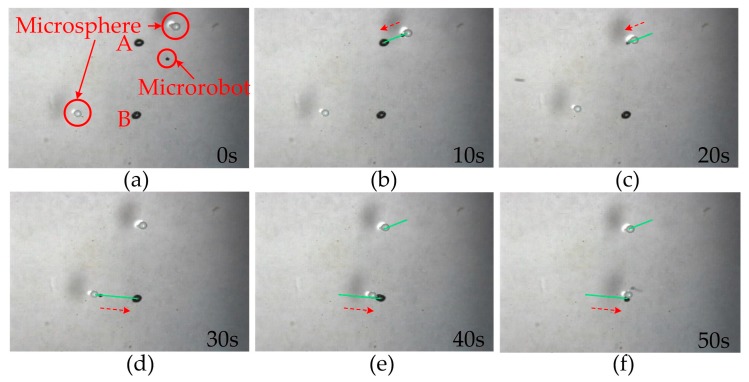
Manipulation of the microrobot with two microspheres. Two microspheres are placed on the liquid surface randomly (**a**). The microrobot is controlled to manipulate the first microsphere to the desired point A automatically (**b**). Then, it accelerates to leave the first microsphere and moves to the second (**c**). The microrobot manipulates the second microsphere (**d**) and automatically moves to point B (**e**). Finally, it leaves the second microsphere at point B (**f**) and moves on. The manipulation procedure takes approximately 50 s.

We can also control the microrobot to manipulate two microspheres. In Figure 10, two microspheres are placed on the liquid surface randomly (a). The microrobot is controlled to manipulate the first microsphere to the desired point A automatically (b). Then, it accelerates to leave the first microsphere and moves to the second (c). The microrobot manipulates the second microsphere (d) and automatically moves to point B (e). Finally, it leaves the second microsphere at point B (f) and moves on. The two microspheres are patterned at point A and B successfully. The manipulation procedure with two microspheres takes approximately 50 s. The experiment results show that the microrobot can drag, place, and pattern the microspheres automatically with high precision.

### 4.5. Manipulating Microgear

**Figure 11:**
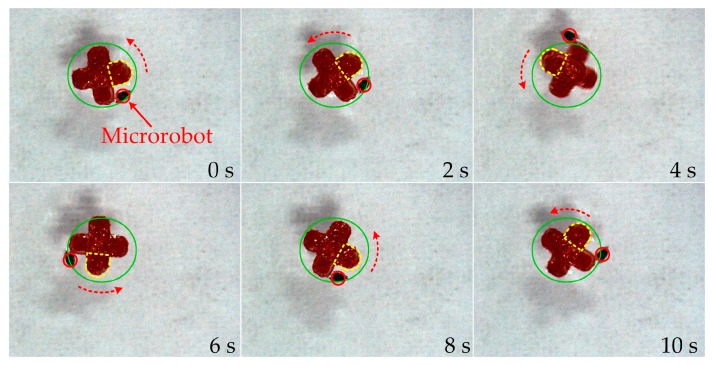
Rotate a gear by the microrobot. The microrobot pulls the gear to rotate by tracking a circular path. The rotational speed of the gear is approximately 6 rpm.

The microrobot can be controlled to manipulate a red PDMS gear with four teeth. The external diameter of the gear is 2.25 mm. When the microrobot contacts the gear, it attaches to the gear as a result of the tensile force. In Figure 11, the microrobot is controlled to do a circular motion by tracking a predefined circular path (green circle). Hence, the microrobot pulls the gear to rotate. One tooth of the gear is stroked in yellow to observe the rotating process easily. The rotational speed of the gear is approximately 6 rpm. The experiment result shows that the microrobot can manipulate microstructures with high precision and flexibility. The manual control method is difficult to achieve rotating the gear. The experiment result also proves the effectiveness of the proposed automatic control method.

## 5. Conclusions

This paper proposed an automatic control method for a microrobot by means of an electromagnetic manipulation system. The microrobot was designed as a thin disk with diameter 600 μm, which is fabricated from a mixture of PDMS and NdFeB magnetic powder. The electromagnetic manipulation system was designed with six position-adaptable electromagnetic coils, which are used to adjust the manipulation region. The microrobot can be controlled to move in the manipulation region automatically by using visual feedback with an expert control algorithm. The positioning accuracy is tested, and the position error ranges from 92 to 293 μm, which is less than half the body size of the microrobot. The effectiveness of the microrobot automatic movement was verified by driving the microrobot to track various paths (rectangular, triangular, hexagonal, square spiral and a complicated customized path). As a micromanipulation tool, the microrobot is used to manipulate microspheres with diameter 1 mm. The experimental results verify that the microrobot can drag, place, and drive the microstructures automatically with high precision. As a result of its small size and high-precision automatic control, the microrobot is expected to be a delicate instrument that could be used for microfluidic control, intravascular survey, drug delivery, and minimal invasive treatment.

## Figures and Tables

**Table 1:** Parameters of electromagnetic coils.

Coils	Coils Turns	Insider Radius/Outsider Radius (mm)	Diameter of Copper Wires (mm)	Resistance (Ω)	Iron Cores
Horizontal	500	55/60	0.51	16	Yes
Vertical	500	55/60	0.51	16	No
